# Airborne lead arsenate chloride (mimetite) microcrystals in ambient air as a potential health hazard

**DOI:** 10.1038/s41598-025-32922-x

**Published:** 2025-12-30

**Authors:** Mariola Jabłońska, Janusz Janeczek, Marzena Rachwał, Wioletta Rogula-Kozłowska

**Affiliations:** 1 Institute of Earth Sciences, University Laboratories of Atmospheric Survey, University of Silesia in Katowice, Będzińska 60, 41-205 Sosnowiec, Poland; 2 Institute of Safety Engineering, Fire University, J. Słowackiego St. 52/54, 01-629 Warsaw, Poland; 3 Institute of Environmental Engineering, Polish Academy of Sciences, M. Curie-Skłodowskiej St. 34, 41-819 Zabrze, Poland

**Keywords:** Airborne mimetite, Air pollution, Topsoil contamination, Dust resuspension, Zabrze (Poland), Chemistry, Environmental sciences

## Abstract

Airborne lead arsenate chloride (mimetite) crystals ranging from sub-micrometer to 10 μm in length and attached to Zn-bearing phase and soot were observed in ambient air in Zabrze, Poland. Mimetite was identified by Raman microspectroscopy and scanning electron microscopy coupled with energy dispersive X-ray spectroscopy. The airborne mimetite, while apparently related to the historic Zn and Pb ore smelting in the region, was collected in a place not directly affected by the smelting. Mimetite commonly occurs in As- and Pb-contaminated topsoil and waste dumps in the region. Six topsoil samples were collected within the small (0.4 km^2^) As, Pb, and Zn geochemical anomaly recorded in the past and located 0.7 km west of the air sampling site to inspect, whether it might have been a source of airborne mimetite. X-ray fluorescence spectroscopy of the samples did not show elevated concentrations of As and Pb. The investigated mimetite microcrystals became airborne most probably due to aeolian entrainment of dust particles from the unspecified location. Calculated health hazard indices suggest a high carcinogenic risk due to prolonged exposure to mimetite in resuspended dust. Mimetite may be a common ambient air pollutant in other places worldwide affected by current or historic emissions from Zn and Pb smelters.

## Introduction

Airborne arsenic (As) and lead (Pb) in particulate matter (PM) are hazardous air pollutants with adverse health impact when inhaled or ingested due to their carcinogenic and mutagenic effects (e.g.^1–5^,^6^,^7^). The natural emission sources of As and Pb include volcanic and geothermal activities, aeolian processes affecting As-, and Pb-bearing weathered rocks, and exudates from vegetation^8,9^. However, As and Pb are emitted to the atmosphere predominantly from anthropogenic activities^9–12^. Coal combustion has the largest share of about 87% in anthropogenic emissions of As followed by non-ferrous metal smelting and the use of pesticides^12^. Mining operations release As-laden particulates through dust emissions^11^. Biomass burning can be an important airborne As source in some regions of the world^10,12^.

Arsenic in air occurs principally in particulate form as inorganic compounds of its trivalent (arsenite) or pentavalent (arsenate) ions with the As(III)/As(V) ratio in the range of 0.06 to 1.42^13^. Speciation of airborne As can be mass size fraction dependent. Tirez et al.^14^ showed the predominance of As(V) in PM with an aerodynamic diameter < 10 μm (PM_10_) in the vicinity of metallurgical plant; whereas, As(III dominated in PM with an aerodynamic diameter < 2.5 μm (PM_2.5_). However, other studies revealed As(V) prevalence over As(III) in both PM_10_ and PM_2.5_) near copper smelter^15^ and in PM_1_ (particulate matter with an aerodynamic diameter < 1 μm) of ambient air^16^. In general, arsenates concentrations in particulates are higher than arsenites (Lewis et al., 2018).

While As in proximity to smelters and roasters occurs predominantly as As_2_O_3_ in particulate form^11^, it can readily oxidize to arsenate. Among arsenate particulates reported from: non-ferrous smelters flue dust^17,18^, fly-ash emitted from the metallurgical plant^14^, weathered fly-ash^19^, and airborne dust from mine tailings^20^ only lead arsenate chloride, Pb_5_(AsO_4_)_3_Cl, naturally occurring as a mineral mimetite, binds both potentially toxic elements. Mimetite is a widespread secondary mineral in oxidizing zone of Pb deposits and typically occurs as a weathering product of galena, PbS^21^.

Precipitation of mimetite has been proposed as an effective method for arsenate ions removal from solutions^22^ due to its low solubility of 9.31 10^–10^ mol/l^23^. Precipitation of mimetite or hydroxymimetite is a natural attenuation mechanism of As in contaminated soils^24,25^. Mimetite is stable under very low concentrations of dissolved Pb(II) and As(V) in the pH range of natural waters, even in the presence of dissolved carbonate and sulfate ions^26^. However, mimetite, if ingested , may easily be dissolved in human gastric fluids making both As and Pb bioaccessible^17,27,28^.

To the authors’ knowledge mimetite has never been encountered in ambient air in places not directly affected by metallurgical or mining activities. In this paper we report the episodic occurrence of mimetite microcrystals in both PM_1_ and total suspended particulates (TSP) collected in ambient air in a city of Zabrze (pron. Zabzhe), S Poland. A possible human health hazard caused by exposure to inhaled mimetite is assessed and the probable sources of airborne mimetite are discussed.

### Site characterization

**Fig. 1:**
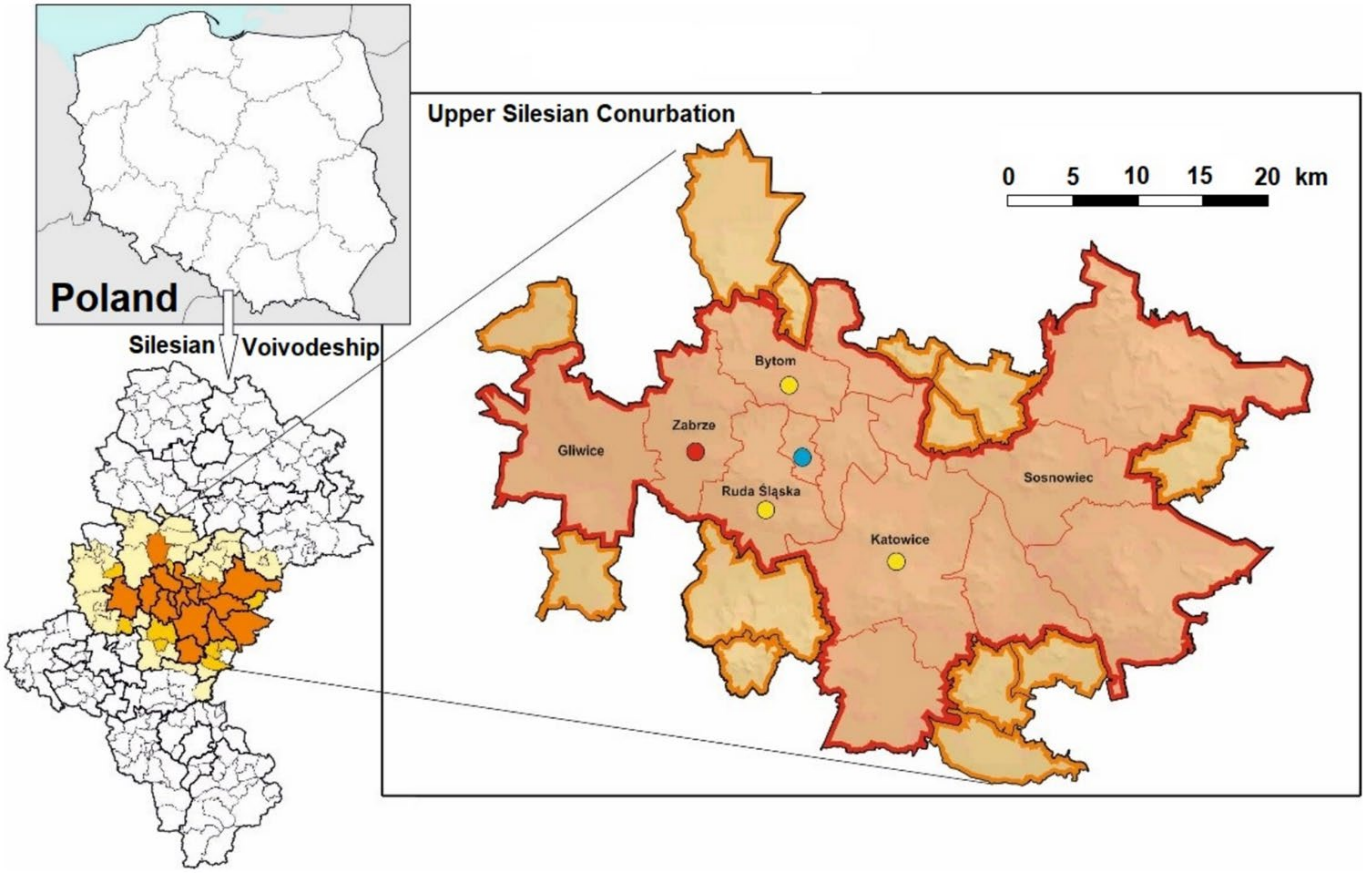
Recorded occurrences of the environmental mimetite in the Upper Silesian Conurbation in: airborne dust particles (red dot; this study); topsoil (yellow dots;^29^), historic Zn-smelter slags (blue dot,^30^).

Samples were collected at the air quality monitoring station in the Zabrze downtown (Fig. 1). Zabrze is an industrial city populated by about 153 thousand inhabitants (as of June 2024) in the western part of the Upper Silesian Conurbation (USC) in the Silesia province (voivodship) of southern Poland. USC is an extended urban area consisting of 14 adjacent cities with the total population of 1.85 million. Together with an additional 15 directly bordering communities, the USC forms one of the most urbanized and industrialized regions of Europe, with ca. 2.5 million people.

The USC is notorious for poor air quality in winter due to widespread residential coal-burning, accounting for 86.9% PM_2.5_, 75.9% PM_10_, and 96.6% of benzo(a)pyrene annual emissions in the region^31^. The USC is a part of the Upper Silesia Industrial Region (USIR) with numerous emission point sources, including: electricity- and heat-generating coal-fired plants, metal manufacturers, coking and chemical plants, and coal mines.

At the beginning of the twentieth century there were 33 Zn and Pb smelters in the USIR^32^. Numerous waste dumps, mine tailings, and the contamination of soils with heavy metals (Zn, Pb, Cd, Cu, Ag) and metalloids (As, Sb) are the legacy of this historical ore mining and smelting^33^. While the regional geochemical background in the USIR for As and Pb is 6 mg/kg and 72 mg/kg, respectively, the maximum contents of these elements determined in 2806 topsoil samples are 5288 mg/kg and 54,940 mg/kg, respectively^33^. Some of As and Pb precipitated in topsoil as mimetite.

Current emissions of As and Pb in the Silesia province are low. Mean annual mass concentrations of Pb in PM_10_ in 2023 ranged from 0.01 to 0.03 μg/m^3^, i.e. well below the permissible limit value of 0.5 μg/m^3^^31^. Mean annual concentrations of As in PM_10_ in 2023 ranged from 0.8 to 1.2 ng/m^3^; whereas, health-based annual target value for the atmospheric As is 6 ng/m^3^^31^.

### Sampling

A total of 22 samples of total suspended particulates (TSP) and 22 samples of PM_1_ were collected on a daily basis from 24 October 2018 to 30 March 2019 at the air quality monitoring station in Zabrze (according to WGS84 coordinate system: 50.3165°N, 18.772237°E). The monitoring station was located in close proximity to a trunk road (approximately 500 m to the north), residential apartment blocks and individual houses to the east, the city center with residential and commercial housing to the south and southeast, and residential apartment blocks and allotments to the west.

For the present study, samples were collected at 6–8 day intervals. Consequently, the initial 24-h samples of PM1 and TSP were collected on 24 October, with subsequent samples collected on 1 November. The present article focuses exclusively on the samples in which mimetite was identified (based on SEM/EDX analyses and confirmed by Raman spectroscopy): namely Z-63, Z-79, and Z-124, collected on December 25th, January 10th, and February 24th, respectively. The nomenclature employed for the samples varied according to whether they were PM1 or TSP; in the former case, the fraction name was placed before the sample name, for example PM1-Z-63, TSP-Z-124.

The collection of samples was conducted in 24-h cycles on 47 mm Whatman QMA quartz filters which application was predicated on their comparatively diminished analytical background in comparison to glass filters. The low-volume impactor Atmoservice PNS3D15/LVS3D, operating at a stabilized flow rate of 2.3 m^3^/h was applied in this study. It was equipped with two distinct separating heads, thereby enabling the retention of particles within a specified diameter range: to the selective collection of PM1 and TSP. The mass concentration of particulate matter was determined by means of a standard gravimetric measurement method in accordance with the^34^ standard and referred to the volume of air passed (μg/m^3^). Prior to and after measurements, the filters were subjected to a conditioning period lasting 48 h at room temperatures of 22 ± 2 °C and relative humidity levels of 38 ± 5%. Thereafter, the filters were weighted.

**Fig. 2:**
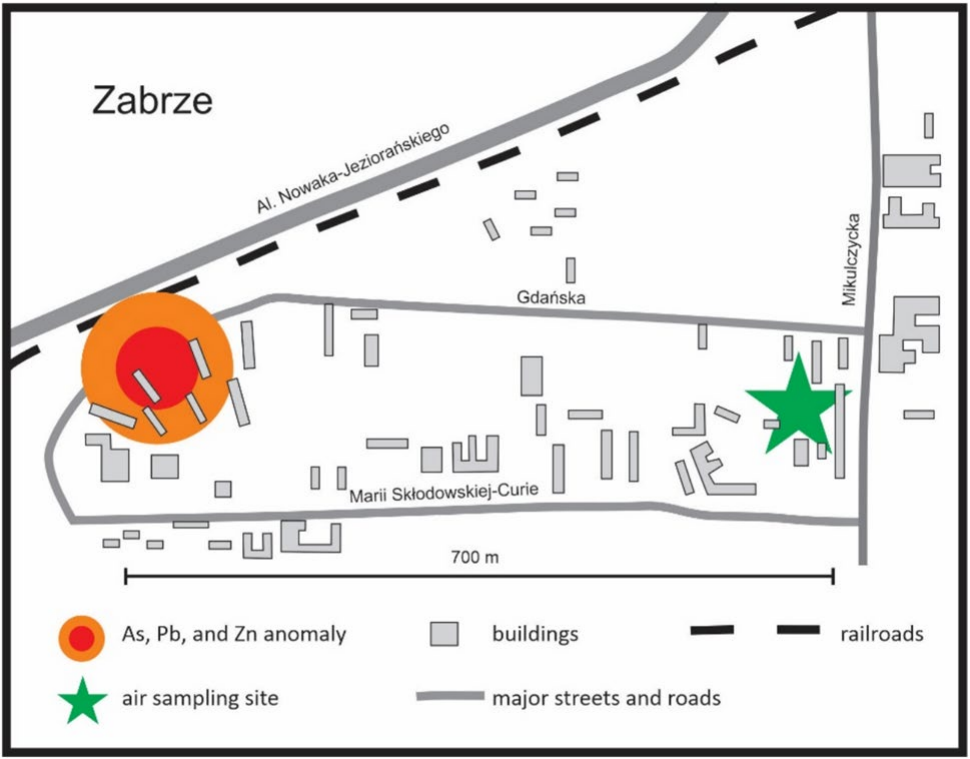
Sketch map of the locations of the As and Pb circular geochemical anomaly reported by Pasieczna et al.^35^ and the air sampling site in Zabrze. Red circle contours topsoil with 320 to 781 ppm As and over 1000 ppm Pb. The top soil marked by the orange rim contained between 90 and 160 ppm As and 500 to 1000 ppm Pb (data from ^35^).

Small (ca. 0.4 km^2^) circular anthropogenic geochemical anomaly of As (781 mg/kg), Pb (> 500 mg/kg), and Zn (> 1000 mg/kg) in topsoil was reported some 0.7 km west of the air sampling site^35^ (Fig. 2). Currently the anomaly is covered by community gardens, lawns, and tall residential buildings. Six topsoil samples (0–20 cm depth) were collected at various spots within the anomaly in January of 2023 by the present authors and analyzed by X-ray fluorescence spectroscopy to check whether the anomaly could be a potential source of windblown As-Pb-rich dust.

## Methods

### As and Pb determination

In order to determine the total content of As and Pb High-Resolution Inductively Coupled Plasma-Mass Spectrometry (HR-ICP-MS, 6100 DRC-e Perkin Elmer, Waltham, MA, USA) was applied. The collected filters were previously digested in a mixture of nitric acid and hydrogen peroxide solution in a microwave digestive system.

### Scanning electron microscopy (SEM) and energy dispersive X-ray (EDX) spectroscopy

Secondary electron (SE) and back-scattered electron (BSE) imaging and elemental analysis of individual atmospheric particles were performed on unpolished polyphasic grains with a scanning electron microscope Quanta 250 coupled to X-ray microanalyzer EDS UltraDry (Thermo Fisher Scientific) operated at 15 kV accelerating voltage. The working distance was 10 mm and spectrum acquisition time 90 s. The electron beam diameter was 5 μm. Chemical composition of mimetite was determined semi-quantitatively by the standardless EDX analysis using the Pathfinder software.

Based on EDX analyses, a semi-quantitative assessment of the phases present in all analysed samples (22 of PM_1_ and 22 of TSP) was performed and categorized as present, common, minor, or rare.

### Raman microspectroscopy

The occurrence of mimetite was confirmed by Raman microspectroscopy using a WITec alpha 300R Confocal Raman Microscope, equipped with solid-state laser, optical objective LD EC Epiplan-Neofluan DIC—100/0.75NA, and a CCD camera. Spectra with the best signal-to-noise ratio were acquired using excitation wavelength of 488 nm at a resolution of 3 cm^−1^ in the range of 100 – 4000 cm^−1^. Each of the accumulated Raman spectra was the sum of 20 short-exposure (3 to 5 s) spectra. Spectra were calibrated with the 520.7 cm^−1^ silicon excitation line.

### X-ray fluorescence (XRF) analysis

Six samples of topsoil collected within the supposed As and Pb geochemical anomaly nearby the sampling site were analyzed in the wavelength dispersion mode using the S8 TIGER Series 2 XRF spectrometer (Bruker, Karlsruhe, Germany) with a 1 kW Rh X-ray tube. The spectrometer is equipped with five crystals (LiF200, PET, XS–55, LIF-220 & Ge) and two detectors (flow and scintillation counter). Six grams of each finely powdered sample was mixed with 1 g of cellulose wax binder and pressed in a die at 12 tons. The pressed pellets were analyzed for 18 minutes in vacuum using the standardless method. The data were calculated by means of a Quant-Express program belonging to the Spectra Plus software suite.

### Health hazard indices

The potential human health hazard associated with environmental exposures to inhaled contaminants were estimated according to the USEPA’s guidance^36^ by calculating hazard quotient (HQ), non-carcinogenic risk assessment concerning neurotoxicity, mutagenicity, developmental and reproductive toxicity and carcinogenic risk (CR)^37^,^38^. The hazard quotient (HQ) was computed as the ratio of the exposure concentration of the inhalable chemical (EC) to a reference concentration (RfC) meaning an estimate of a continuous inhalation exposure to the human population that is likely to be without an appreciable risk of deleterious effects during a lifetime. RfC values for As and Pb are 1.5 × 10^–5^ and 0.8 × 10^–2^ mg/m^3^, respectively^36^:

1 $$HQ \, = \, EC/\left( {RfC \, \times 1000 \, \mu g/mg} \right)$$

The carcinogenic risks (CR) were computed by multiplying the exposure concentration of the inhalable chemical (EC) by the inhalation unit risk (IUR):

2 $$CR \, = \, EC \, \times \, IUR$$

Values of IUR are as follows: 4.3 × 10^–3^ and 1.2 × 10^–5^ µg/m^3^ for As and Pb, respectively^39^,^40^. EC was calculated using the following equations^36^:

3 $$EC \, \left( {\mu g/m^{3} } \right) \, = \, \left( {C \, \times \, ET \, \times \, EF \, \times \, ED} \right)/AT,$$

where C is As and Pb contents (µg/m^3^), ET – exposure time (6 h/day), EF – exposure frequency (330 days/year), ED—exposure duration (70 years) and AT—averaging time (70 × 365 days/year × 24 h/day)^41^. To assess the health risk, it was assumed that exposure occurs when staying outdoors. It was assumed that on average a person spends about 6 h outside buildings during the day, on average 3 days a month he does not leave buildings, hence, he is at risk of inhaling outdoor air pollutants for about 330 days a year.

## Results

### Mineral inventory of PM_1_ and TSP

In total 53 minerals and other phases have been observed by SEM/EDX in all of PM samples. They varied in quantity and frequency of the occurrence. Those observed in all or almost all (> 90%) samples included soot (common), Fe-oxides (mostly hematite identified by the Raman spectroscopy, and ranging from common in one sample of TSP to rare), fly ash (minor to absent), and carbonaceous matter (C + N) other than soot (rare, but present in all samples). The unspecified compound with an elemental composition of Ba, Cl, F, and O occurred in 86% of PM_1_ samples and was particularly abundant in 3 samples and minor in 8 others, including the one with mimetite. That compound was subordinate or rare in 68% of TSP samples. No attempt was made to further characterize that phase. Other frequently observed phases included Ca sulfate, chlorides of Na, K, Pb, and Zn, barite, and Zn-spinel.

Mimetite-bearing samples were observed on three days (December 12, 2018, January 10 and February 24, 2019) with dominant westerly winds and high concentrations of As and Pb in PM_1_ and TSP (Table 1).

**Table 1:** Particulate matter (PM_1_ and TSP), As and Pb mass concentrations, and meteorological data for sampling days of the mimetite-bearing dust in Zabrze, S Poland.

Sample number	Sampling day	PM_1_ TSP μg/m^3^		As [ng/m^3^] PM_1_ TSP		Pb [μg/m^3^] PM_1_ TSP		wind direction	wind speed m/s	T °C	P hPa	RH %	Rain mm
Z-63	Dec. 25	15.03	38.17	166.34	130.57	6904.57	9155.56	NW, WNW,W	1.96	0.4	993	95	0.15
Z-79	Jan. 10	20.54	24.92	287.42	162.26	5028.30	5466.54	NNW, NNE,N	2.04	-1.8	987	95	0.25
Z-124	Feb. 24	15.48	55.95	299.63	307.75	8486.43	9170.81	WNW,S, WSW, W	0.70	0.2	1006	66	0.00

Mimetite was abundant in one sample of PM_1_; whereas, in two others, it occurred in noticeable quantities. Phases associated with mimetite are listed in Table 2. Besides mimetite, the following Pb-bearing phases occurred in 15 PM_1_ and 8 TSP samples: chloride, carbonate, oxide, and sulfate.

**Table 2:** Mineral inventory of mimetite-bearing PM_1_ and TSP samples.

Minerals and other phases	PM_1_	TSP
mimetite	xxx—x	x
Ba-Cl-F-O	xxx—n	x
soot	xx	x
fly ash	xxx—n	xxx
“talc”	xx—x	xx
Zn-carbonate	xx—n	n
C + N	xx—x	x
Ca-sulfate	xx—n	n
Na, K, Ca, Mg-sulfates	xx—n	xx
barite	x—n	x
hematite	x	n
NaCl, KCl	x	n

Xxx–common, xx–minor, x–rare, n–absent.

### Mimetite characterization

**Fig. 3:**
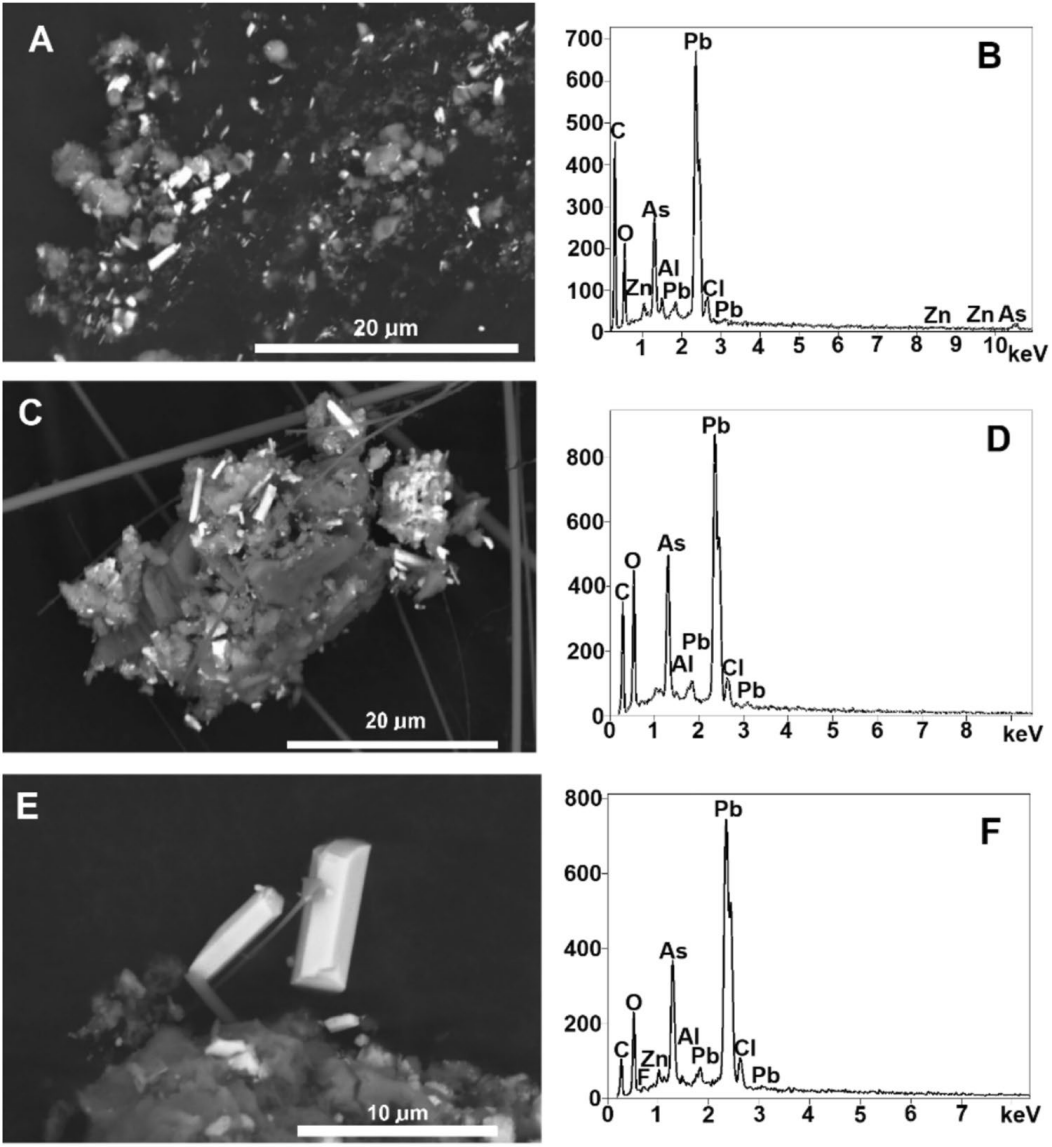
SEM images and EDX spectra of airborne mimetite from Zabrze. (**A**) mimetite crystals (bright) and Zn-bearing phase (grey) attached to soot aggregates in PM_1_; (**B**) EDX spectrum of mimetite from (**A**). (**C**) mimetite crystals in PM_1_ on the Zn-bearing phase ; (**D**) EDX spectrum of mimetite; (**E**) single crystals of mimetite (note the hexagonal symmetry of the smaller crystal) and (**F**) EDX spectrum of the larger (right) crystal.

Mimetite crystals occur on the unspecified Zn-bearing phase (Fig. 3) in coarse aggregated particles or are attached to soot aggregates (Fig. 2). Prismatic and acicular crystals of mimetite are up to 8 μm in length (Fig. 3). A width to length ratio ranges from 0.07 to 0.39. The lowest width to length ratio is higher than typical for mimetite acicular crystals (0.02–0.03)^42^. Morphologically, mimetite crystals in PM_1_ closely resemble acicular crystals of mimetite precipitates obtained through desorption of As(V) and Pb(II) from goethite (α-FeOOH)^43^. The acicular habit of the mimetite crystals is indicative of fast crystal growth, regardless of its origin, i.e., either from vapor deposition or from aqueous solution.

In addition to Pb, As, and Cl, the mimetite EDX spectra show weak peaks of Zn from the unspecified Zn-bearing phase and Al (Fig. 3). The source of Al has not been determined.

**Fig. 4:**
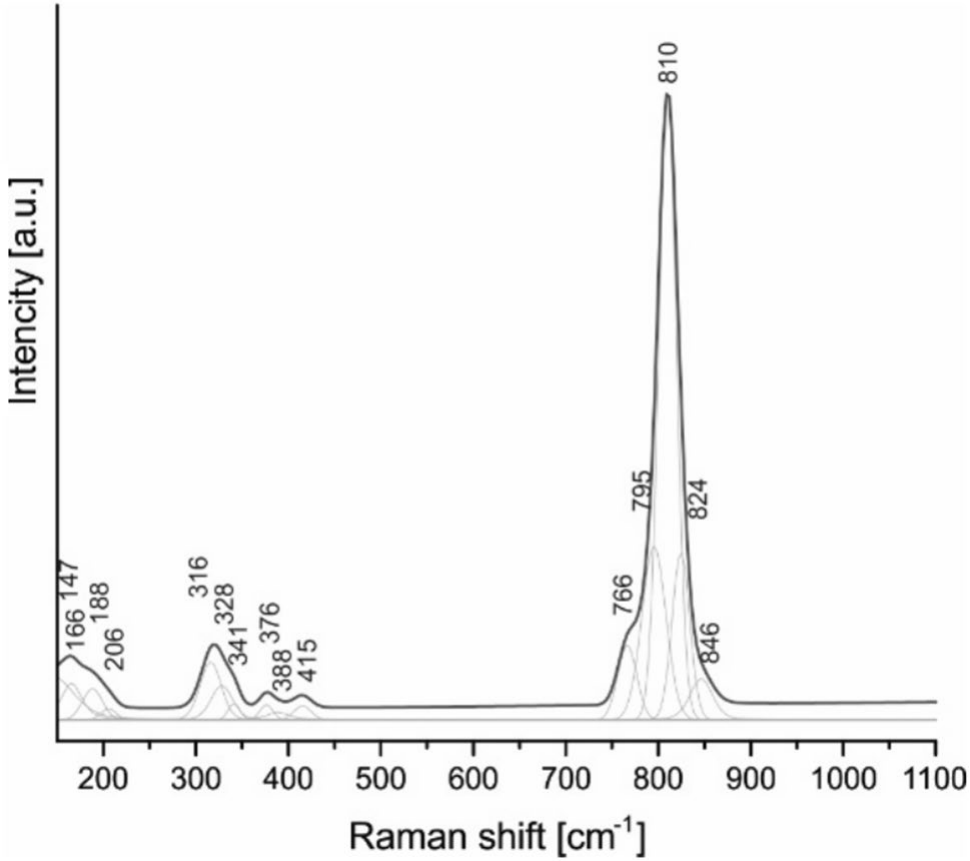
Raman spectrum of airborne mimetite from Zabrze.

The occurrence of mimetite in samples investigated during this study was confirmed by Raman microspectroscopy. The obtained spectrum (Fig. 4) is similar to mimetite spectra published by Bajda^23^, Frost et al.^44^ and Szełęg et al.^45^. The most prominent band in the Raman spectrum of mimetite is attributed to symmetric stretching vibrations^44^. In the spectrum of the airborne mimetite that band occurs at 810 cm^−1^; while wavenumbers reported in the literature range from 820 cm^−1^^46^ through 816 cm^−1^^47^, 812–813 cm^−1^^23^,^44^ and 809 cm^−1^^48^ to 805 cm^−1^^45^. Giera et al.^49^ reported the symmetric stretching vibrations band at 811 cm^−1^ for the synthetic Pb_10_(AsO_4_)_6_(OH)_2_.

Chemical composition of the investigated mimetite (Table 3) suggests the lack of the anionic and cationic substitutions at the detection level of the EDAX spectrometer. Zinc content in the EDX analyses varies from 0 to 3.38 wt.%, and is most probably from the Zn-carbonate. The deficit of Cl in EDX analyses may result from OH partial substitution for Cl leading to the composition of Pb_10_(AsO_4_)_6_(Cl_2 – x_ OH_x/2_)^50^.

**Table 3:** Semi-quantitative elemental compositions (wt.%) of airborne mimetite from Zabrze and ideal mimetite, Pb_5_(AsO_4_)_3_Cl.

	TSP-Z-124	PM-Z-63–1	PM-Z-63–2	Pb_5_(AsO_4_)_3_Cl
As_2_O_5_	21.23	22.09	20.93	23.17
PbO	75.52	75.72	74.73	74.99
ZnO	0.78	0.00	2.25	-
Cl	2.47	2.19	2.09	2.38
-O = Cl_2_	0.56	0.49	0.47	0.54
Total	99.44	99.51	99.53	100.00

### Potential health hazard of airborne mimetite

Health hazard indices: HQ and CR (Table 4) enabled a health risk assessment. If hazard quotient exceeds unity the potential health effects may occur. Otherwise (HQ < 1), it is assumed that the risk is at an acceptable level. The cancer risk below 1 × 10^−6^ is negligible, whereas a risk level of 1 × 10^−4^ or greater is considered serious^36^,^40^. The calculated HQ below unity (Table 4) suggests negligible non-carcinogenic risk. However, CR calculated for inhaled As and Pb in PM_1_ and TSP is > 1 × 10^−6^ (Table 4); thus, the impact of airborne mimetite on human health may be significant. The CR index for Pb is two orders lower than for As. Even if these calculations are oversimplified, they confirm previously raised concerns over the environmental threat to the local populace caused by unprotected contaminated topsoil and waste dumps vulnerable to windblown resuspension of toxic material.

**Table 4:** Hazard quotients (HQ) and carcinogenic risks (CR) caused by inhalation of As and Pb in TSP and PM_1_.

	HQ			CR		
Sample number	As	Pb	ΣHQ	As	Pb	ΣCR
PM-Z-63	2.49 × 10^–2^	4.96 × 10^–4^	2.54 × 10^–2^	1.61 × 10^–6^	4.76 × 10^–8^	1.66 × 10^–6^
PM-Z-79	3.68 × 10^–2^	3.78 × 10^–4^	3.72 × 10^–2^	2.37 × 10^–6^	3.63 × 10^–8^	2.41 × 10^–6^
PM-Z-124	5.77 × 10^–2^	5.27 × 10^–4^	5.83 × 10^–2^	3.72 × 10^–6^	5.06 × 10^–8^	3.78 × 10^–6^
Mean	3.98 × 10^–2^	4.67 × 10^–4^	4.03 × 10^–2^	2.57 × 10^–6^	4.48 × 10^–8^	2.61 × 10^–6^
TSP-Z-63	3.32 × 10^–2^	6.91 × 10^–4^	3.39 × 10^–2^	2.14 × 10^–6^	6.63 × 10^–8^	2.21 × 10^–6^
TSP-Z-79	4.28 × 10^–2^	4.16 × 10^–4^	4.32 × 10^–2^	2.76 × 10^–6^	3.99 × 10^–8^	2.80 × 10^–6^
TSP-Z-124	5.20 × 10^–2^	5.60 × 10^–4^	5.25 × 10^–2^	3.35 × 10^–6^	5.38 × 10^–8^	3.41 × 10^–6^
Mean	4.27 × 10^–2^	5.56 × 10^–4^	4.32 × 10^–2^	2.75 × 10^–6^	5.34 × 10^–8^	2.81 × 10^–6^

## Discussion

Coal combustion and Zn-Pb-ore processing and smelting have been major emission source categories of mimetite-forming elements, i.e., As, Pb, and Cl in the Silesian province^51^. The content of As in the Upper Silesian coals ranges from 0.1 to 28.9 μg/kg and Pb content ranges from 1.2 to 89.0 μg/kg averaging at 5.2 μg/kg^52^. The seasonality in As concentration in PM_1_ was observed at the air-quality monitoring station in Zabrze. In winter, total (3.26 ng/m^3^) and soluble (1.86 ng/m^3^) As concentrations were 1.2 and 3 times higher, respectively, than in summer^16^. In winter, mass concentrations of water soluble As(V) and As(III) were 55% and 3.5%, respectively of the total As; whereas, in summer mass concentrations of As(V) decreased to 23%, while As(III) increased to 6.7%. Nocoń & Rogula-Kozłowska^16^ concluded that fossil fuel combustion was the major source of PM_1_-bound As, at least in winter. That conclusion is apparently supported by the present study’s observation of the spatial association of airborne mimetite and soot. However, the co-occurrence of mimetite and Zn-carbonate suggests rather non-ferrous metallurgical processes as a source of mimetite.

One of the largest and modern European Zn and Pb smelter is operating some 25 km NE of the sampling site. The As content in PbS collected in dust from electrostatic precipitator and other processing stages of Zn and Pb smelting in that smelter ranges from 0.03 to 1.06 wt.% As_2_O_3_^53^. Lead (73.27 wt.% PbO) and Zn (15.22 wt.% ZnO) are major constituents of the electrostatic precipitator dust from the sinter unit of the smelter accounting for 88 wt.% of the dust with As_2_O_3_ content of 0.56 wt%^53^.

There are numerous waste dumps, mine tailings, and contaminated soils around historic sites of Zn and Pb mining, ore processing and smelting in the USC, which are potential sources of windblown dust. Moreover, materials from waste dumps have been widely used for road construction and landfilling. Large, regional-scale geochemical Pb and As anomalies related to waste dumps and contaminated soils around former Zn and Pb mining and smelting sites occur at a distance of 10 to 14 km NE to SE of the sampling site^54,55^. Cadmium-rich mimetite (1.23 wt% CdO) intergrown with barite and associated with Zn arsenate was observed as a minor secondary constituent of Zn-smelting slag from a dump within the As-Pb anomaly in the city of Świętochłowice (Fig. 1) ^30^.

Airborne mimetite reported in this paper was observed on the days with dominant westerly winds (Table 1); therefore, both, the operating Zn and Pb smelter to the NE and regional geochemical anomalies to the E of Zabrze were rather unlikely source of windblown dust collected at the sampling site.

Results of the XRF analyses of samples collected within the geochemical anomaly located some 0.7 km west of the air sampling site in Zabrze^35^ showed the maximum contents of Zn and Pb equal to 800 mg/kg and 350 mg/kg, respectively; while As was below the detection limit (10 mg/kg). These values are much lower than reported by Pasieczna et al.^35^. Most probably, the anomaly is now hidden under the cover of the delivered soil,hence it is the unlikely emission source of mimetite-bearing windblown dust.

Abundant acicular mimetite microcrystals (< 20 μm) amounting up to 45% of a total of 200 mineral grains inspected in each of topsoil samples around three sites of historic metallurgical plants in the USC were reported by Nadłonek^29^ (Fig. 1). The contents of As and Pb in the mimetite-bearing topsoil range from a few to > 10 000 mg/kg and there is a significant correlation between both elements (op. cit.). Mimetite occurs in association with galena, cerrussite (PbCO_3_), barite (BaSO_4_), anglesite (PbSO_4_), Ca-sulfate, Zn-silicate (possibly hemimorphite), and Fe-, Zn-hydroxides. Mimetite may have formed either as a sublimate in flue gases in smelters or as a precipitate from pore solutions in topsoil^17,18,24,25^. For instance, mimetite has not been observed in As-enriched flue dust originating from a Cu smelter; instead, mimetite crystallized in the same flue dust incubated in various forest and grassland soils^46^. Pore solutions in the mimetite-bearing topsoils of Upper Silesia are either pH neutral or alkaline^29^, i.e., favorable for mimetite crystallization and stability^26^,^23,24^.

The morphology and size of mimetite crystals in topsoil around three sites of historic metallurgical plants in the USC^29^ are identical to the airborne mimetite reported in this study. We conclude that the observed airborne mimetite was most likely windblown from the mimetite-bearing material; hence it did not form in the ambient air as the secondary aerosol. We noticed a red debris typical of metallurgical waste, used for the stabilization of ground at the construction of a housing estate nearby the air sampling site. There is a possibility that the debris may have contained mimetite microcrystals. Unfortunately, sampling of the debris was not possible.

The spatial association of airborne aggregates of mimetite and Zn-carbonate with soot suggests that the resuspended mimetite-bearing dust was adsorbed to the surface of soot particles in air. Soot is ubiquitous in the USC ambient air, particularly in winter as a result of inefficient coal combustion for domestic purposes; whereas, in summer soot from vehicular exhaust is predominant.

Due to its relatively low solubility in water, mimetite effectively immobilizes As and Pb in soils and water solutions. However, mimetite solubility increases in solutions containing organic acids^56–58^ and, as already mentioned in the Introduction, mimetite is easily soluble in gastric fluid^17,27,28^. Therefore, airborne mimetite and other arsenates may deliver bioaccessible As, which is classified as class 1 carcinogenic agent^38^ to humans through both respiration and ingestion of airborne dust. There is evidence of As pulmonary carcinogenicity not only caused by inhalation but also by ingestion of As-bearing particles^11^.

Preliminary calculations indicate that a single mimetite crystal (Fig. 3E) contains approximately 0.02 ng As. Consequently, 300 microcrystals per cubic meter of air contain 6 nanograms of arsenic, which corresponds to the annual health-based target value for arsenic in air^59^. The deleterious effects of airborne mimetite on human health are corroborated by health hazard indices, particularly the CR calculated for inhalable As and Pb in PM1 and TSP which is greater than 1 × 10^−6^ (Table 4). The results suggest that the widespread use of historic, unrecycled mining and metallurgical wastes as engineering materials may pose a health risk in locations remote from the waste source.

## Conclusions

While our findings are limited to a single locality and to relatively small amount of airborne mimetite microcrystals, results reported in this paper seem to be valid to any region with present or past non-ferrous metals smelting activities, not necessarily in proximity of the metallurgical plants. Mimetite is an environmental sink for As and Pb in contaminated topsoil; however, it can be windblown resuspended in relatively high quantities of microcrystals posing real threat of inhalation or ingestion by people. The widespread use of historic mine and metallurgical unrecycled waste as an engineering material, as it is the case in the studied area, may pose health hazard in places distant from the waste source. The prolonged exposure to mimetite in the inhaled air does not cause a non-carcinogenic risk to human health. However, there is a high carcinogenic risk, especially due to As in resuspended dust.

## Data Availability

Data obtained during this study are available on request.

## References

[1] Basu, A., Mahata, J., Gupta, S. & Girl, A. K. Genetic toxicology of a paradoxical human carcinogen, arsenic: A review. *Mutat. Res.* **488**, 171–194. 10.1016/S1383-5742(01)00056-4 (2001). 11344043 10.1016/s1383-5742(01)00056-4

[2] Bellinger, D. C. Very low lead exposures and children’s neurodevelopment. *Curr. Opin. Pediatr.* **20**(2), 172–177. 10.1097/MOP.0b013e3282f4f97b (2008). 18332714 10.1097/MOP.0b013e3282f4f97b

[3] Sanchez-Rodas, D., De la Campa, A. S., Oliveira, V. & De la Rosa, J. Health implications of the distribution of arsenic species in airborne particulate matter. *J. Inorg. Biochem.* **108**, 112. 10.1016/j.jinorgbio.2011.11.023 (2012). 22196019 10.1016/j.jinorgbio.2011.11.023

[4] Zhang, R., Wilson, V. L., Hou, A. & Meng, G. Source of lead pollution, its influence on public health and the countermeasures. *Int. J. Health Animal Sci. Food Saf.* **2**, 18–31. 10.13130/2283-3927/4785 (2015).

[5] Yin, X. et al. Arsenic accumulation and speciation of PM25 and relevant health risk assessment in Jinan China. *Pol. J. Environ. Stud.* **26**, 949–954. 10.15244/pjoes/66714 (2017).

[6] Zhang, L., Gao, Y., Wu, S, Zhang, S., Smith, K.R., Yao, X., & Gao, H. 2020 Global impact of atmospheric arsenic on health risk: 2005 to 2015. PNAS, 117(25):13975–13982 10.1073/pnas.2002580117 10.1073/pnas.2002580117 PMC7322006 32513708

[7] Rahaman, M. S. et al. Environmental arsenic exposure and its contribution to human diseases, toxicity mechanism and management. *Environ. Pollut.* **289**, 117940. 10.1016/j.envpol.2021.117940 (2021). 34426183 10.1016/j.envpol.2021.117940

[8] Matschullat, J. Arsenic in the geosphere—A review. *Sci. Total Environ.* **249**(1–3), 297–312. 10.1016/s0048-9697(99)00524-0 (2000). 10813460 10.1016/s0048-9697(99)00524-0

[9] Meharg, A. A. & Meharg, C. The pedosphere as a sink, source, and record of anthropogenic and natural arsenic atmospheric deposition. *Environ. Sci. Technol* **55**, 7757–7769. 10.1021/acs.est.1c00460 (2021). 34048658 10.1021/acs.est.1c00460

[10] Lin, Y.-C. et al. Enhancements of airborne particulate arsenic over the subtropical free troposphere: impact of southern Asian biomass burning. *Atmos. Chem. Phys.* **18**, 13865–13879. 10.5194/acp-18-13865-2018 (2018).

[11] Martin, R., Dowling, K., Pearce, D., Sillitoe, J. & Florentine, S. Health effects associated with inhalation of airborne arsenic arising from mining operations. *Geosciences* **4**, 128–175. 10.3390/geosciences4030128 (2014).

[12] Vishwakarma, Y. K., Tiwari, S., Mohan, D. & Singh, R. S. A review on health impacts, monitoring and mitigation strategies of arsenic compounds present in air. *Cleaner Eng. Technol.* **3**, 100115. 10.1016/j.clet.2021.100115 (2021).

[13] Lewis, A. S., Reid, K. R., Pollock, M. C. & Campleman, S. L. Speciated arsenic in air: Measurement methodology and risk assessment considerations. *J. Air Waste Manag. Assoc.* **62**(1), 2–17. 10.1080/10473289.2011.608620 (2012). 22393805 10.1080/10473289.2011.608620

[14] Tirez, Z. et al. Speciation of inorganic arsenic in particulate matter by combining HPLC/ICP-MS and XANES analyses. *J. Anal. At. Spectrom.* **30**, 2074–2088. 10.1039/C5JA00105F (2015).

[15] Gonzales-Castanedo, J. et al. Arsenic species in atmospheric particulate matter as tracer of the air quality of Donana natural park (SW Spain). *Chemosphere* **119**, 1296–1303. 10.1016/j.chemosphere.2014.09.093 (2015). 25460775 10.1016/j.chemosphere.2014.09.093

[16] Nocoń, K. & Rogula-Kozłowska, W. Speciation of arsenic: A case study of PM1 in Zabrze. *SN Appl. Sci.* **1**, 450. 10.1007/s42452-019-0456-x (2019).

[17] Ettler, V. et al. Oral bioaccessibility of metal(loid)s in dust materials from mining areas of northern Namibia. *Environ. Int.* **124**, 205–215. 10.1016/j.envint.2018.12.027 (2019). 30654327 10.1016/j.envint.2018.12.027

[18] Schindler, M., Santosh, M., Dotto, G., Silva, L. F. O. & Hochella, M. F. Jr. A review on Pb-bearing nanoparticles, particulate matter and colloids released from mining and smelting activities. *Gondwana Res.*10.1016/j.gr.2021.07.011 (2021).

[19] Ettler, V., Mihaljevič, M., Šebek, O., Valigurová, R. & Klementová, M. Differences in antimony and arsenic releases from lead smelter fly ash in soils. *Geochemistry* **72**(S4), 15–22. 10.1016/j.chemer.2012.01.004 (2012).

[20] Bailey, A. S., Jamieson, H. E. & Radková, A. B. Geochemical characterization of dust from arsenic-bearing tailings, Giant Mine. *Canada. Appl. Geochem.* **135**, 105119. 10.1016/j.apgeochem.2021.105119 (2021).

[21] Keim, M. & Markl, G. Weathering of galena: Mineralogical processes, hydrogeochemical fluid path modeling, and estimation of the growth rate of pyromorphite. *Am. Mineral.* **100**, 1584–1594. 10.2138/am-2015-5183 (2015).

[22] Bajda, T., Szmit, E., & Manecki, M.: Removal of As(V) from solutions by precipitation of mimetite, Pb_5_(AsO_4_)_3_Cl, in: Environmental Engineering edited by: Pawłowski, L., Dudzińska, M., Pawłowski, A., Taylor & Francis: London, 119–124, 2007.

[23] Bajda, T. Solubility of mimetite Pb_5_(AsO_4_)_3_Cl at 5–55°C. *Environ. Chem.* **7**, 268–278. 10.1071/EN10021 (2010).

[24] Gutiérrez-Ruiz, M. E., Ceniceros-Gómez, A. E., Villalobos, M., Romero, F. & Santiago, P. Natural arsenic attenuation via metal arsenate precipitation in soils contaminated with metallurgical wastes: II. Cumulative evidence and identification of minor processes. *Appl. Geochem.* **27**, 2204–2214. 10.1016/j.apgeochem.2012.02.021 (2012).

[25] Thouin, H. et al. Influence of environmental changes on the biogeochemistry of arsenic in soil polluted by the destruction of chemical weapons : A mesocosm study. *J. Sci. Tot. Environ*10.1016/j.scitotenv.2018.01.158 (2018). 10.1016/j.scitotenv.2018.01.158 29426144

[26] Magalhães, M. C. F. & Silva, M. C. M. Stability of lead(II) arsenates. *Monatshefte für Chemie/Chemical Monthly* **134**, 735–743. 10.1007/s00706-002-0581-9 (2003).

[27] Mullins, M. J. P. & Norman, J. B. Solubility of metals in windblown dust from mine waste dump sites. *Appl. Occup. Environ. Hyg.* **9**(3), 218–223. 10.1080/1047322X.1994.10388301 (1994).

[28] Plumlee, G. S. & Morman, S. A. Mine wastes and human health. *Elements* **7**, 399–404. 10.2113/gselements.7.6.399 (2011).

[29] Nadłonek W.: Heavy metals and metalloids (Zn, Pb, Cd, As, Sb) in soils and wastes in areas of Zn-Pb smelting (in Polish). PhD thesis. University of Silesia, Sosnowiec, 2020.

[30] Bril, H. et al. Secondary phases from the alteration of a pile of zinc-smelting slag as indicators of environmental conditions: An example from Świętochłowice, Upper Silesia. *Poland. Can. Mineral.* **46**, 1235–1248. 10.3749/canmin.46.5.1235 (2008).

[31] Annual evaluation of the air quality in the Silesia voivodship in 2023. Main Inspectorate of the Environment Protection. Regional Department of the Environmental Monitoring. Katowice, Poland (in Polish), 2024.

[32] Cabała, J., Janeczek, J. & Kowalczyk, A.: Lead in the environment. Narrations of the Shoah – Special Issue, 10.31261/NoZ.2021.DHC.09, 2021.

[33] Pasieczna, A., Konon, A. & Nadłonek, W. Sources of anthropogenic contamination of soil in the upper Silesian Agglomeration (southern Poland). *Geol. Q.* **64**(4), 988–1003. 10.7306/gq.1564 (2020).

[34] PN-EN 12341:2014–07: Ambient air - Standard gravimetric measurement method for the determination of the PM10 or PM2,5 mass concentration of suspended particulate matter.

[35] Pasieczna, A. (ed.), Fajfer, J., Strzemińska, K.: Detailed geochemical map of Upper Silesia at scale of 1:25 000, map sheet Zabrze M-34–62-B-a, The Polish Geological Institute - National Research Institute, Warsaw, Poland, 2016.

[36] USEPA: US Environmental Protection Agency. Part F. Supplemental guidance for inhalation risk assessment. In Risk Assessment Guidance for Superfund Volume I: Human Health Evaluation Manual; Office of Superfund Remediation and Technology Innovation: Potomac Yards, VA, USA, p. 68, 2009.

[37] Zheng, J. et al. Levels, sources, markers and health risks of heavy metals in PM2.5 over a typical mining and metallurgical city of Central China. *Aerosol Sci. Eng.* **2**, 1–10. 10.1007/s41810-017-0018-9 (2017).

[38] IARC: International Agency for Research on Cancer. Agents Classified by the IARC Monographs: https://monographs.iarc.fr/list-of-classifications (last access: 31 August 2024), 2020.

[39] USEPA. Integrated Risk Information System (IRIS); National Center for Environmental Assessment: Washington, DC, USA, 2002.

[40] Ramírez, O., Sánchez de la Campa, A. M., Sánchez-Rodas, D. & de la Rosa, J. D. Hazardous trace elements in thoracic fraction of airborne particulate matter: assessment of temporal variations, sources, and health risks in a megacity. *Sci. Total Environ.* **710**(136344), 2020. 10.1016/j.scitotenv.2019.136344 (2020). 10.1016/j.scitotenv.2019.136344 31923687

[41] Rachwał, M., Wawer, M., Jabłońska, M., Rogula-Kozłowska, W. & Rogula-Kopiec, P. Geochemical and mineralogical characteristics of airborne particulate matter in relation to human health risk. *Minerals* **10**, 866. 10.3390/min10100866 (2020).

[42] Thiéry, V. Characterization of fibrous mimetite. *Microsc. Microanal.* **20**, 596–601. 10.1017/S143192761301413X (2014). 24548472 10.1017/S143192761301413X

[43] Kleszczewska-Zębala, A., Manecki, M., Bajda, T., Rakovan, J. & Borkiewicz, O. J. Mimetite formation from goethite-adsorbed ions. *Microsc. Microanal.* **22**, 698–705. 10.1017/S1431927616000829 (2016). 27329315 10.1017/S1431927616000829

[44] Frost, R. L., Bouzaid, J. M. & Palmer, S. The structure of mimetite, arsenian pyromorphite and hedyphane—A Raman spectroscopic study. *Polyhedron* **26**, 2964–2970. 10.1016/j.poly.2007.01.038 (2007).

[45] Szełęg, E., Janeczek, J., Juroszek, R. & Danila, M. Mimetite and polymineralic mimetite-pyromorphite-vanadinite single crystals from the Sowie Mts Poland. *Mineralogia* **55**, 48–59. 10.2478/mipo-2024-0005 (2024).

[46] Jarošíková, A. et al. Transformation of arsenic-rich copper smelter flue dust in contrasting soils: A 2-year field experiment. *Environ. Pollut.* **237**, 83–92. 10.1016/j.envpol.2018.02.028 (2018). 29477118 10.1016/j.envpol.2018.02.028

[47] Bartholomäi, G. & Klee, W. E. The vibrational spectra of pyromorphite, vanadinite and mimetite. *Spectrochim. Acta A* **34**(7), 831–843. 10.1016/0584-8539(78)80038-5 (1978).

[48] Levitt, S. R. & Condrate, R. A. Sr. Vibrational spectra of lead apatites. *Am. Miner.* **55**(9–10), 1562–1575 (1970).

[49] Giera, A. et al. Arsenate substitution in lead hydroxyl apatites: A Raman spectroscopic study. *Spectrochim. Acta, Part A* **152**, 370–377. 10.1016/j.saa.2015.07.015 (2016). 10.1016/j.saa.2015.07.015 26232581

[50] Baikie, T. et al. Crystal chemistry of mimetite, Pb_10_(AsO_4_)_6_Cl_1.48_O_0.26_, and finnemanite, Pb_10_(AsO_3_)_6_Cl_2_. *Acta Crystallogr.*10.1107/S0108768107066402 (2008). 10.1107/S0108768107066402 18204209

[51] Jabłońska, M. & Janeczek, J. Identification of industrial point sources of airborne dust particles in an urban environment by a combined mineralogical and meteorological analyses: A case study from the Upper Silesian conurbation. *Poland. Atmos. Pollut. Res.* **10**, 980–988. 10.1016/j.apr.2019.01.006 (2019).

[52] Parzentny, H. & Róg, L. Evaluation of certain petrographic, physico-chemical, and geochemical parameters of coals quality in the paralic series of the Upper Silesian coal basin and attempt to determine the correlations among them (in Polish). *Miner. Resour. Manag.* **33**(1), 51–76. 10.1515/gospo-2017-0004 (2017).

[53] Nowińska, K. & Adamczyk, Z. Effect of galena contained in dust from Zn–Pb metallurgical processes on environment. *Environ. Earth Sci.* **80**, 294. 10.1007/s12665-021-09594-7 (2021).

[54] Lis, J. & Pasieczna, A. Pb-Zn-Cd geochemical anomalies in soils of Upper Silesia. *Polish Geol. Rev.* **45**(2), 182–189 (1997).

[55] Pasieczna, A., & Konon, A.: Detailed geochemical map of Upper Silesia 1:25 000, map sheet: Piekary Śląskie. The Polish Geological Institute - National Research Institute, Warsaw, Poland, 2021.

[56] Bajda, T. Dissolution of mimetite Pb5(AsO4)3Cl in low-molecular-weight organic acids and EDTA. *Chemosphere* **83**(11), 1493–1501. 10.1016/j.chemosphere.2011.01.056 (2011). 21345478 10.1016/j.chemosphere.2011.01.056

[57] Ceci, A., Kierans, M., Hillier, S., Persiani, A. M. & Gadd, G. M. Fungal bioweathering of mimetite and a general geomycological model for lead apatite mineral biotransformations. *Appl. Environ. Microbiol.* **81**, 955–4964. 10.1128/AEM.00726-15 (2015). 10.1128/AEM.00726-15 PMC4495193 25979898

[58] Turek, P., Bajda, T. & Manecki, M. Dissolution of mimetite Pb5(AsO4)3Cl in malic acid solutions. *Mineralogia* **45**(1–2), 3–12. 10.2478/mipo-2014-0001 (2014).

[59] Air quality standards: https://www.eea.europa.eu/themes/air/air-quality-concentrations/air-quality-standards 31.09.2022, 2022, September 31, last access: 20 June, 2024,

